# Supplementary material for: Electron-Transferring Flavoprotein and Its Dehydrogenase Required for Fungal Pathogenicity in Arthrobotrys oligospora
Source: Int J Mol Sci. 2024 Oct 11;25(20):10934. doi: 10.3390/ijms252010934 (PMC11507118; doi:10.3390/ijms252010934)
Supplement: Supplementary file 1 [file ijms-25-10934-s001.zip › ijms-3230269-supplementary.pdf]

Supplementary Materials

Supplementary Figures

Fig S1. Characterization of ETF-α protein in *A. oligospora*. Phylogenetic tree of the ETF-α orthologs in different fungi using the neighbor-joining method. The numbers represent bootstrap values.

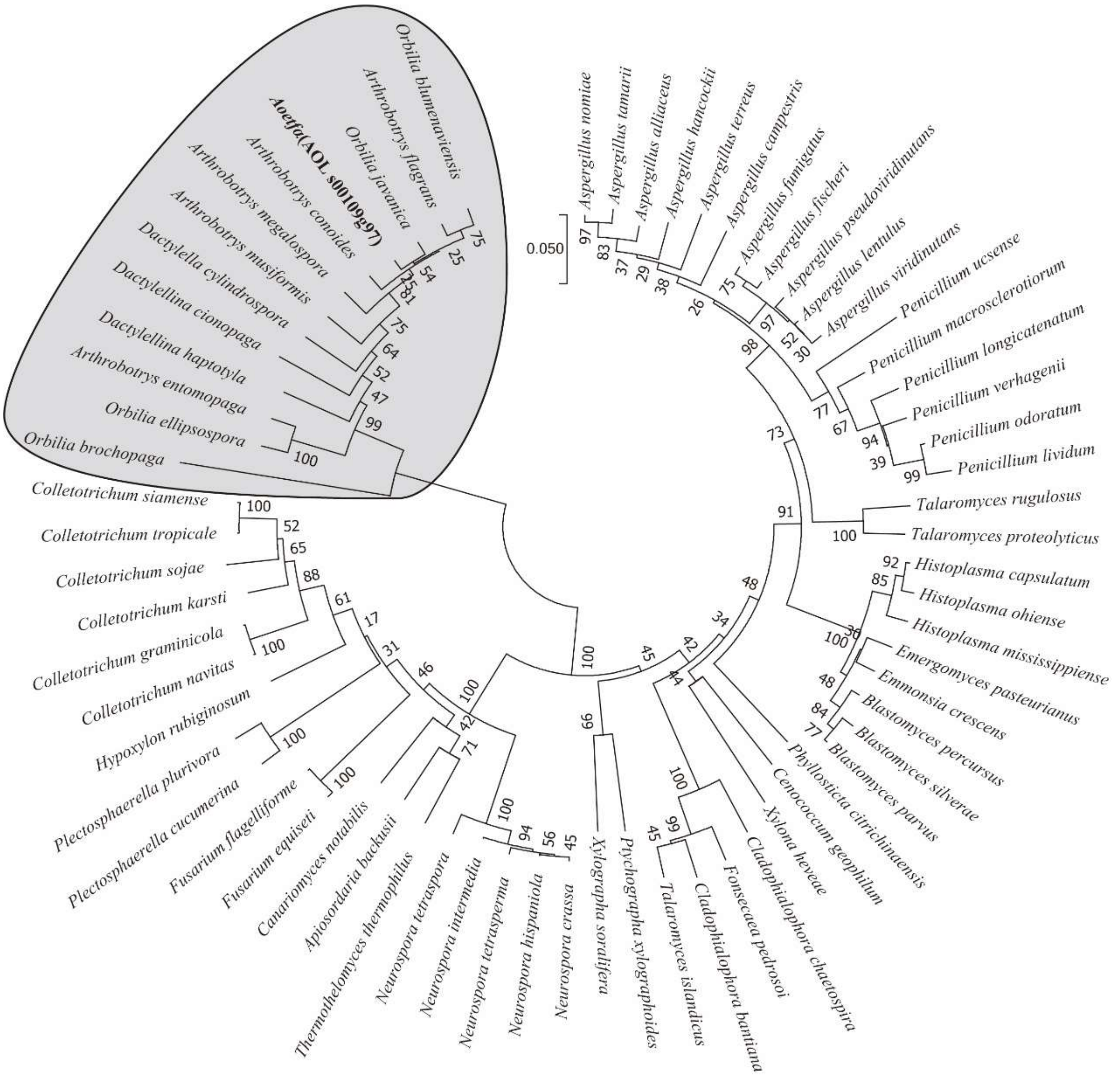

Fig S2. Characterization of ETF-β protein in *A. oligospora*. Phylogenetic tree of the ETF-β orthologs in different fungi using the neighbor-joining method. The numbers represent bootstrap values.

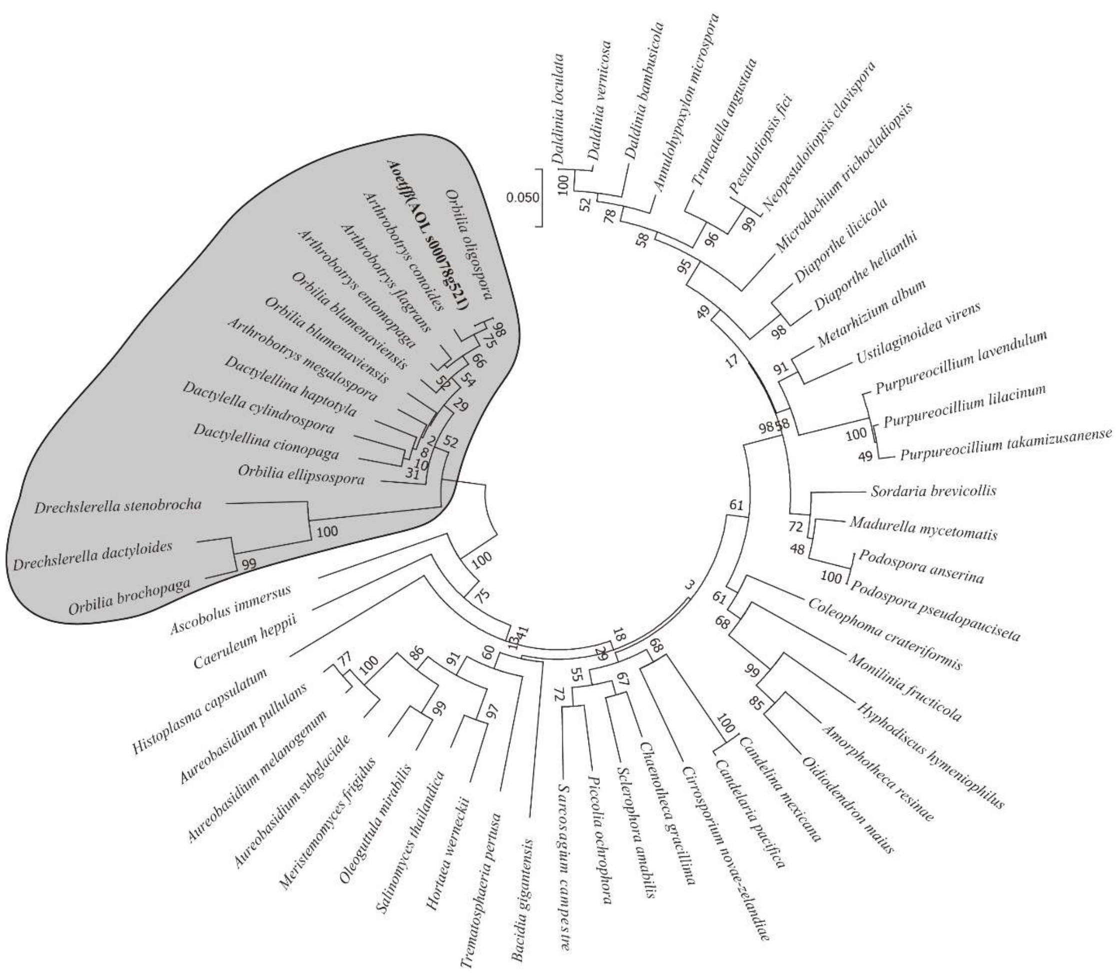

Fig S3. Characterization of ETFdh protein in *A. oligospora*. Phylogenetic tree of the ETFdh orthologs in different fungi using the neighbor-joining method. The numbers represent bootstrap values.

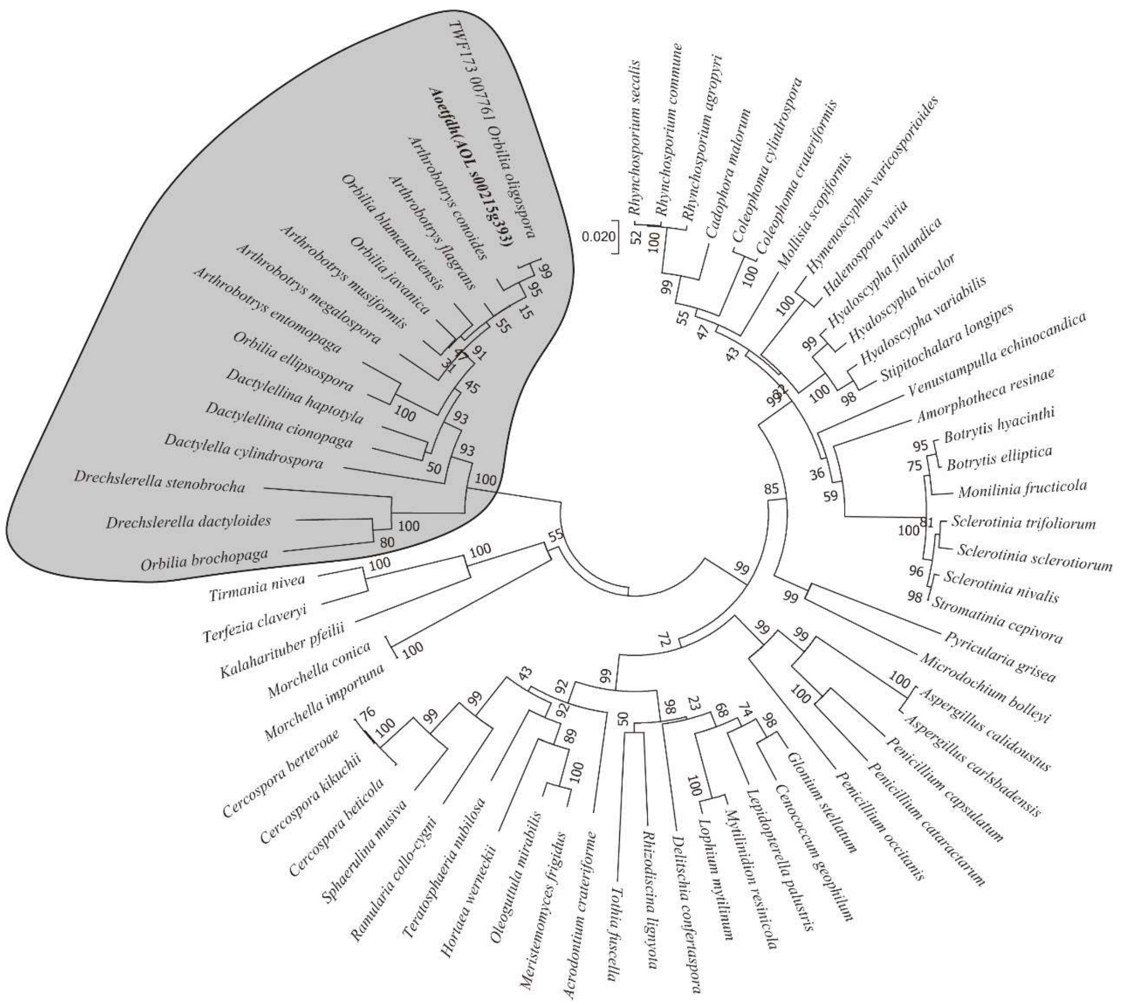

Fig S4. Targeted *Aoetf* gene deletion and growth phenotype. (A) Confirmation of *Aoetfα*, *Aoetfβ*, *Aoetfdh* deletion mutant by PCR strategy. (B) Growth trends of WT, *ΔAoetfα*, *ΔAoetfβ*, and *ΔAoetfdh* strains on PDA, CMY, TG, and TYGA media.

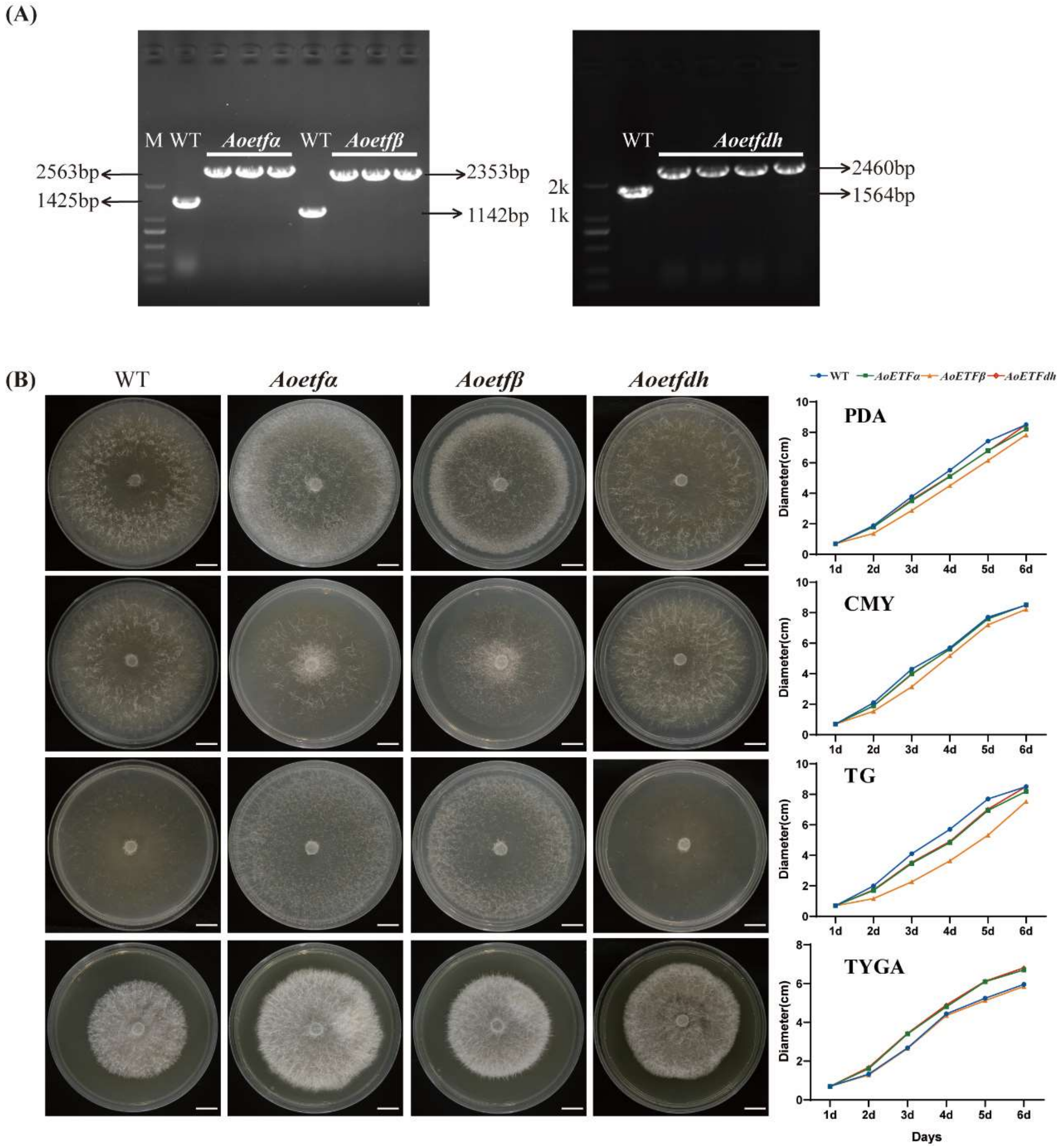

Fig S5. WT and *ΔAoetfβ* metabolomics and exosome analysis. (A) In the metabolomics volcano plot, cetraxate is significantly upregulated. (B) The NTA analysis and TEM image of WT exosome. (C) The NTA analysis and TEM image of *ΔAoetfβ* exosome (Scale bar: 100 nm).

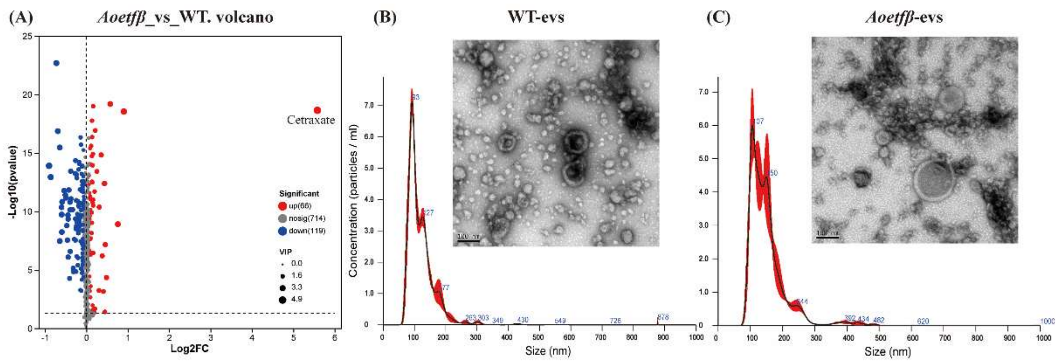

Supplementary tables

Table S1 List of primers for gene disruption in this study

Primers	Sequence
**Amplify the *Aoetfα*, *Aoetfβ* and *Aoetfdh* gene 5' flank**	
109g97-5F	5'-aattcggatcttccagagatGGAACATGGACAGCAAAG -3'
109g97-5R	5'-atccttctttCGTTGTTCGAAGAGTGCTAG-3'
78g521-5F	5'-aattcggatcttccagagatatCGTGCCCGTAGTAGATAG-3'
78g521-5R	5'-atccttctttCGAGGATTCTAAGGGTCG -3'
215g393-5F	5'-aattcggatcttccagagatTATCACGTACAGTCCGTTG -3'
215g393-5R	5'-atccttctttGATTCCTCATCTGTGGCG -3'
**Amplify the *Aoetfα*, *Aoetfβ* and *Aoetfdh* gene 3' flank**	
109g97-3F	5'-tcatcttctgGCACCTATCTTCCAAGTTG-3'
109g97-3R	5'-ttcaactgccgttcgacgatGCGGAATTACCTGCATATTC-3'
78g521-3F	5'-tcatcttctgGAAGGAGCTTGGTGCCTTG-3'
78g521-3R	5'-ttcaactgccgttcgacgatTCTCAGTATCTGGCGACC-3'
215g393-3F	5'-tcatcttctgCTGTCCAGCAGGTGTATAC-3'
215g393-3R	5'-ttcaactgccgttcgacgatGTAACATACCTGTCCCAG-3'
**Amplify the *hph* cassette**	
109g97-HF	5'-tcgaacaacgAAAGAAGGATTACCTCTAAAC-3
109g97-HR	5'-agataggtgcCAGAAGATGATATTGAAGGAG-3'

78g521-HF	5'-agaatcctcgAAAGAAGGATTACCTCTAAAC-3'
78g521-HR	5'-aagctccttcCAGAAGATGATATTGAAGGAG-3'
215g393-HF	5'-atgaggaatcAAAGAAGGATTACCTCTAAAC-3'
215g393-HR	5'-tgctggacagCAGAAGATGATATTGAAGGAG-3'
**Verify the transformants**	
109g97-F	5'-TCTACATCTTGACCGCAGC -3'
109g97-R	5'-CTGTGGCATCAGTCGACTTG-3'
78g521-F	5'-GCTCCTCTCCATCTACGAAC-3'
78g521-R	5'-CGCGTGATTGGAGACTTCAA-3'
215g393-F	5'-TTGAACCACATGTTCTCGACG-3'
215g393-R	5'-GGGAAGGTGCCTTGATATCAC-3'

Table S2 List of primers for RNAi in this study

Primers	Sequence
**Amplify the *ETF*α gene RNAi fragment**	
F27D4 RNAi F	5'- GGCGGCCGCTCTAGAACTAGTGAACAGCACTCTTGTTGTCG -3'
F27D4 RNAi R	5'- TCCACGCGTCACGTGGCTAGCCCTTCGCATCTTCGCATC -3'
**Amplify the *ETF*β gene RNAi fragment**	
F23C8 RNAi F	5'- GGCGGCCGCTCTAGAACTAGTGATTCTCGTCGGAGTCAAG -3'
F23C8 RNAi R	5'- TCCACGCGTCACGTGGCTAGCAGTGTTGCGTAAGCTACGC -3'
